# Risk prediction models for maternal mortality: A systematic review and meta-analysis

**DOI:** 10.1371/journal.pone.0208563

**Published:** 2018-12-04

**Authors:** Kazuyoshi Aoyama, Rohan D’Souza, Ruxandra Pinto, Joel G. Ray, Andrea Hill, Damon C. Scales, Stephen E. Lapinsky, Gareth R. Seaward, Michelle Hladunewich, Prakesh S. Shah, Robert A. Fowler

**Affiliations:** 1 Department of Anesthesia and Pain Medicine, The Hospital for Sick Children, Toronto, ON, Canada; 2 Institute of Health Policy, Management and Evaluation, University of Toronto, Toronto, ON, Canada; 3 Department of Obstetrics and Gynaecology, Division of Maternal-Fetal Medicine, Mount Sinai Hospital, Toronto, ON, Canada; 4 Department of Critical Care Medicine, Sunnybrook Health Science Center, Toronto, ON, Canada; 5 Keenan Research Centre of the Li Ka Shing Knowledge Institute of St. Michael’s Hospital, Toronto, ON, Canada; 6 Department of Obstetrics and Gynecology, St. Michael’s Hospital, Toronto, ON, Canada; 7 Department of Critical Care Medicine, Mount Sinai Hospital and University Health Network, Toronto, ON, Canada; 8 Kidney Care Centre, Sunnybrook Health Science Center, Toronto, ON, Canada; 9 Departments of Paediatrics, Mount Sinai Hospital, Toronto, ON, Canada; The University of Warwick, UNITED KINGDOM

## Abstract

**Purpose:**

Pregnancy-related critical illness leads to death for 3–14% of affected women. Although identifying patients at risk could facilitate preventive strategies, guide therapy, and help in clinical research, no prior systematic review of this literature exploring the validity of risk prediction models for maternal mortality exists. Therefore, we have systematically reviewed and meta-analyzed risk prediction models for maternal mortality.

**Methods:**

Search strategy: MEDLINE, EMBASE and Scopus, from inception to May 2017.

Selection criteria: Trials or observational studies evaluating risk prediction models for maternal mortality.

Data collection and analysis: Two reviewers independently assessed studies for eligibility and methodological quality, and extracted data on prediction performance.

**Results:**

Thirty-eight studies that evaluated 12 different mortality prediction models were included. Mortality varied across the studies, with an average rate 10.4%, ranging from 0 to 41.7%. The Collaborative Integrated Pregnancy High-dependency Estimate of Risk (CIPHER) model and the Maternal Severity Index had the best performance, were developed and validated from studies of obstetric population with a low risk of bias. The CIPHER applies to critically ill obstetric patients (discrimination: area under the receiver operating characteristic curve (AUC) 0.823 (0.811–0.835), calibration: graphic plot [intercept—0.09, slope 0.92]). The Maternal Severity Index applies to hospitalized obstetric patients (discrimination: AUC 0.826 [0.802–0.851], calibration: standardized mortality ratio 1.02 [0.86–1.20]).

**Conclusions:**

Despite the high heterogeneity of the study populations and the limited number of studies validating the finally eligible prediction models, the CIPHER and the Maternal Severity Index are recommended for use among critically ill and hospitalized pregnant and postpartum women for risk adjustment in clinical research and quality improvement studies. Neither index has sufficient discrimination to be applicable for clinical decision making at the individual patient level.

## Introduction

Pregnancy- and peri-partum-related critical illness occurs at a frequency of 0.7 to 7.6 cases per 1,000 live births in developed countries [1,2], and leads to death for 3–14% of affected women [1,3,4]. Determination of the risk of a woman becoming critically ill or dying is helpful to better anticipate and possibly prevent serious illness and to guide therapeutic decision-making. In clinical research, groups of characteristics that together predict an outcome can be used to help account for differences between patients, when you wish to estimate the influence of some new factor on a clinical outcome such as death [5].

A number of risk prediction models have been developed for outpatients, hospitalized patients, and those who are critically ill. The simplified acute physiology score (SAPS) [6], acute physiology and chronic health evaluation score (APACHE I, II, III, IV) [7], the mortality prediction model (MPM) [8,9], and sequential organ failure assessment (SOFA) scores [10] were originally designed to predict mortality in a general adult intensive care unit (ICU) populations.

These and other prediction models have been applied to pregnant and postpartum women, either in the ICU or in a general ward; however, their performance characteristics have generally not been determined among pregnant and postpartum women [11]. Within obstetrics, a limited number of risk prediction models have been developed for specific obstetric conditions (e.g. preeclampsia, postpartum hemorrhage) [12,13]. Optimal prediction models for unselected, broad cohorts of pregnant and postpartum patients have not been well summarized and previous reviews have concluded that existing *comorbidity* indices have modest predictive ability for obstetric patients [14–16]. While risk prediction models developed from non-pregnant and postpartum populations have been adopted in clinical research for obstetric patients [11], they may have important limitations due to a combination of unique conditions leading to pregnancy-related critical illness and/or death—the typically young age of pregnant patients, and physiological changes specific to pregnancy that may be different from other patient populations. Previously published studies show that non-specific risk prediction models tend to overestimate mortality when applied to pregnant and postpartum women [14,17]. There is no prior systematic review of this literature exploring the validity of risk prediction models for mortality among pregnant and postpartum critically ill women admitted to acute care hospitals.

Therefore, we aimed to systematically review and meta-analyze risk prediction models for maternal mortality in hospitalized and critically ill pregnant and postpartum women.

## Methods

This meta-analysis was conducted on the basis of a guideline for the systematic review of prediction models [18]. The results were reported following the Preferred Reporting Items for Systematic Reviews and Meta-Analyses protocols (PRISMA-P) 2015 statement [19]. This systematic review was registered at PROSPERO (CRD42017070424).

### Criteria for considering studies for this review

#### Type of studies

We included clinical trials, cohort and case-control studies. Case-reports, case-series, reviews and editorials were excluded.

*Participants*. Participants were hospitalized pregnant and postpartum women (to 6 weeks after delivery) in acute care hospitals. Patients in outpatient clinics or emergency rooms were excluded.

#### Index models

Prediction models derived from general hospitalized pregnant and postpartum populations or from critically ill patient populations (e.g. SAPS, APACHE, MPM and SOFA)[6–10]. Models focusing on only specific diagnoses (e.g. preeclampsia, postpartum hemorrhage) were excluded due to their limited generalizability to all obstetric patients [12,13]. We excluded indices that focused only upon pre-existing comorbidity indices as we have previously investigated their predictive performance in obstetric populations [16].

#### Primary outcome of index models

Maternal mortality (death during pregnancy and up to 42 days after delivery or termination of pregnancy).

### Search methods for identification of studies

#### Electronic search

MEDLINE, EMBASE (OvidSP) and Scopus were searched systematically for eligible studies, from their inception to May 2017 (S1 File), containing three sets of terms reflecting the research question: the models (index risk prediction models), the target condition (maternal critical illness or death), and the patient population (pregnant and postpartum women). Known models were included as a key word in a broader search strategy (S1 File) [6–10]. No language restriction was made.

#### Searching other resources

In addition to identified articles retrieved from electronic databases, a citation search was performed in Web of Science to identify other articles that cited the identified articles above. A manual search was conducted from the reference lists of the Web of Science identified articles. Lastly, experts (SL, JGR) in the field were contacted to identify unpublished studies or studies that may not have been captured in MEDLINE, EMBASE and Scopus.

### Data collection and analysis

#### Selection of studies

Inclusion criteria: 1) study reports performance of a mortality risk prediction model 2) while pregnant or within 42 days of delivery or termination of the pregnancy; and, 3) among patients admitted to an acute care hospital.

Exclusion criteria: 1) study design was a case-report, case-series, reviews or editorials; 2) patient population was pregnant or postpartum women with a specific diagnosis (e.g. only pre-eclamptic women); or, 3) indices including only pre-existing comorbid conditions.

The two independent reviewers (KA, RD) scanned the titles and abstracts of every record retrieved to determine whether the article was relevant, according to the above eligibility criteria. The full text of potentially eligible articles was then retrieved. The reference lists of retrieved articles were also searched for additional citations. Two reviewers (KA, RD) independently assessed and determined the eligibility of studies. Disagreement was resolved by discussion and, when necessary, a third reviewer (RAF) assisted in adjudicating a final decision.

#### Data extraction and management

Reviewers used standardized, piloted data forms to independently extract data from all eligible studies. Each data element was compared between primary and secondary reviewers. Any discrepancies were resolved by discussion or adjudication by the third reviewer. Each study was described by general information (title, journal, year, publication status and study design [prospective or retrospective]), descriptors (number of included patients, age, country, subgroups, type of risk prediction model, and stated purpose of the model), reference information (clinical follow-up, mortality rate) and descriptors relevant for assessing the fitness of the model for its intended use: 1) Discrimination—the area under the receiver operating characteristic curve (AUC) or the equivalent c-statistic with 95% confidence interval (CI) or standard error (SE); and, if the AUC or c-statistic was not reported, other operational statistics such as sensitivity and specificity or positive and negative predictive values were recorded when available; 2) Calibration—information on the predicted versus observed mortality ratio is presented as the Standardized Mortality Ratio [SMR] (i.e. observed mortality divided by predicted mortality where SMR < 1 reflects an overestimation of the outcome and SMR > 1 reflects underestimation of the outcome) and goodness-of-fit statistics (e.g. Hosmer-Lemeshow [H-L] goodness-of-fit test) [20]. The corresponding author of the original study was contacted to provide missing data.

We used a recently developed reporting system of prediction models in systematic reviews (Transparent Reporting of a multivariable prediction model for Individual Prognosis Or Diagnosis (TRIPOD) [21,22] and extracted 22 data components for each study in the form of a TRIPOD checklist.

#### Assessment of methodological quality

**Fig 1:**
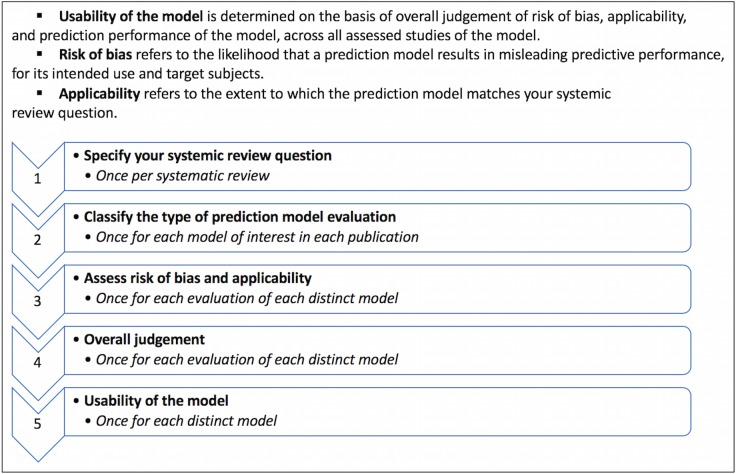
Box summary of PROBAST (Prediction model study Risk Of Bias Assessment Tool).

There is no single standard for the assessment of quality for prediction or prognostic studies[23,24]. However, PROBAST (Prediction model study Risk Of Bias Assessment Tool), a new tool for assessing the methodological quality of risk prediction models was employed [25][Fig 1]. The usability (an actionable recommendation) of a risk prediction model is determined in the following manner[25]. First, the risk of bias and any concerns of applicability *(whether the model fits the research question*: *i*.*e*. *what is the most reliable and best-validated risk adjustment and outcome prediction tool for hospitalized pregnant and postpartum women*?*)* of the model to the intended patient population are noted. Second, the model’s predictive performance (i.e. discrimination and calibration) is considered. Although PROBAST does not specify how good discrimination and calibration should be, generally AUCs higher than or equal to 0.8 for evaluating discrimination are good and an SMR of approximately 1.0 for evaluating calibration is considered as excellent. Calibration can be described in other forms as well—such as graphic plots or according to the Hosmer-Lemeshow statistic. Third, if a risk prediction model has a low risk of bias and low concern about applicability, and is accompanied by good predictive performance, we conclude that the model is quite “usable” (i.e. usability = “Yes”). If studies lack assessment of either discrimination or calibration but are judged to be at low risk of bias and with minimal concerns of applicability, then we have designated usability as “maybe” (a modified definition from PROBAST). However, even when models with low concerns of applicability are applied, subtleties of the original population for model development should still be considered. For example, a model developed from either an obstetric or a non-obstetric population may perform less well if applied to a different population. as might a model developed primarily for critically ill patients or non-critically ill patients because data elements often differ substantially in each setting.

#### Statistical analysis, data synthesis and meta-analysis

The performance of each index was reported as per the original study using the AUC or c-statistic with 95% confidence intervals (CI) or standard error (SE) for discrimination, and standardized mortality ratio or the H-L goodness-of-fit test statistic for calibration [21,22,25]. A meta-analysis was performed if at least three studies evaluating a prediction model were available and reported on the AUC with 95% CIs or SE, or if the AUC could be calculated [18]. The AUC was pooled on the logit scale and the standard errors of the logit transformed AUC were derived from equations previously summarized [18]. We then summarized the AUC using the inverse variance method random-effects model, estimated with restricted maximum likelihood and Hartung-Knapp-Sidik-Jonkman adjustment to generate 95% CIs [18,26].

Clinical heterogeneity across included studies was assessed by examining details of participants and baseline characteristics. The main sources of heterogeneity we expected to encounter related to differences in patients’ baseline characteristics, care delivery and outcomes. The I^2^ statistic was used to explore statistical heterogeneity, defined as moderate when I^2^ = 50–74% and high for I^2^ ≥75%. We planned that if there were more than 10 studies assessing a distinct prediction model for meta-analyses, funnel plots would be drawn to assess the possibility of publication bias [27]. Statistical computations were undertaken using R version 3.4.0 (Free Software Foundation) with R package (meta).

#### Sensitivity and subgroup analyses

Only one sensitivity analysis was pre-planned and included a sub-set of studies, restricted to include those of higher methodological quality (i.e. low risk of bias). One *post-hoc* sensitivity analysis was conducted according to mortality rate: low (under 1%); moderate (to 10%); high to 20%); and very high (greater than 20%).

Two *post-hoc* subgroup analyses were conducted, to investigate high heterogeneity across the eligible studies. One was to separate the all studies into high-income countries, low-income countries or mixed income-countries. The other was to divide the studies into study setting: ICUs or obstetric general wards.

## Results

**Fig 2:**
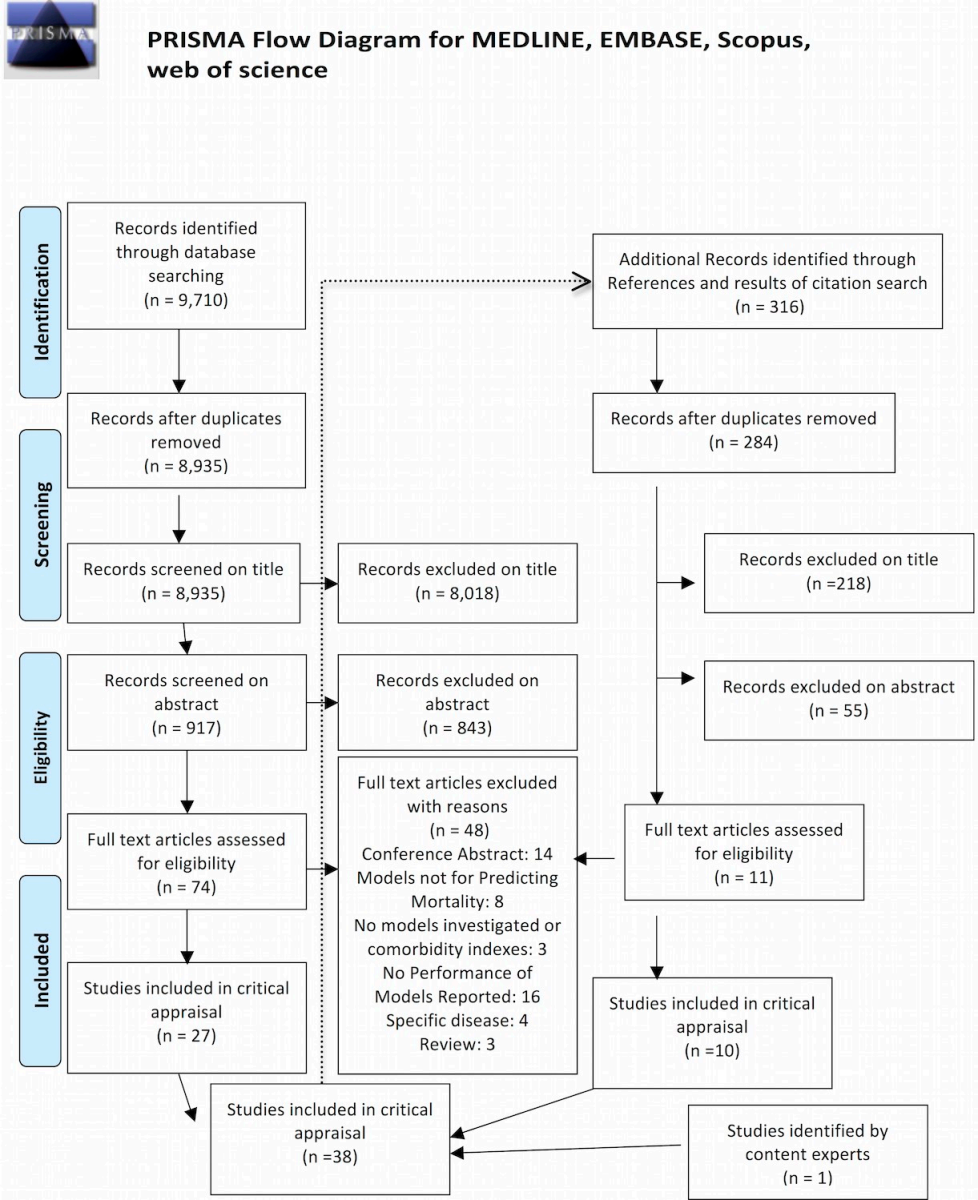
Study selection process (PRISMA flow).

**Fig 3:**
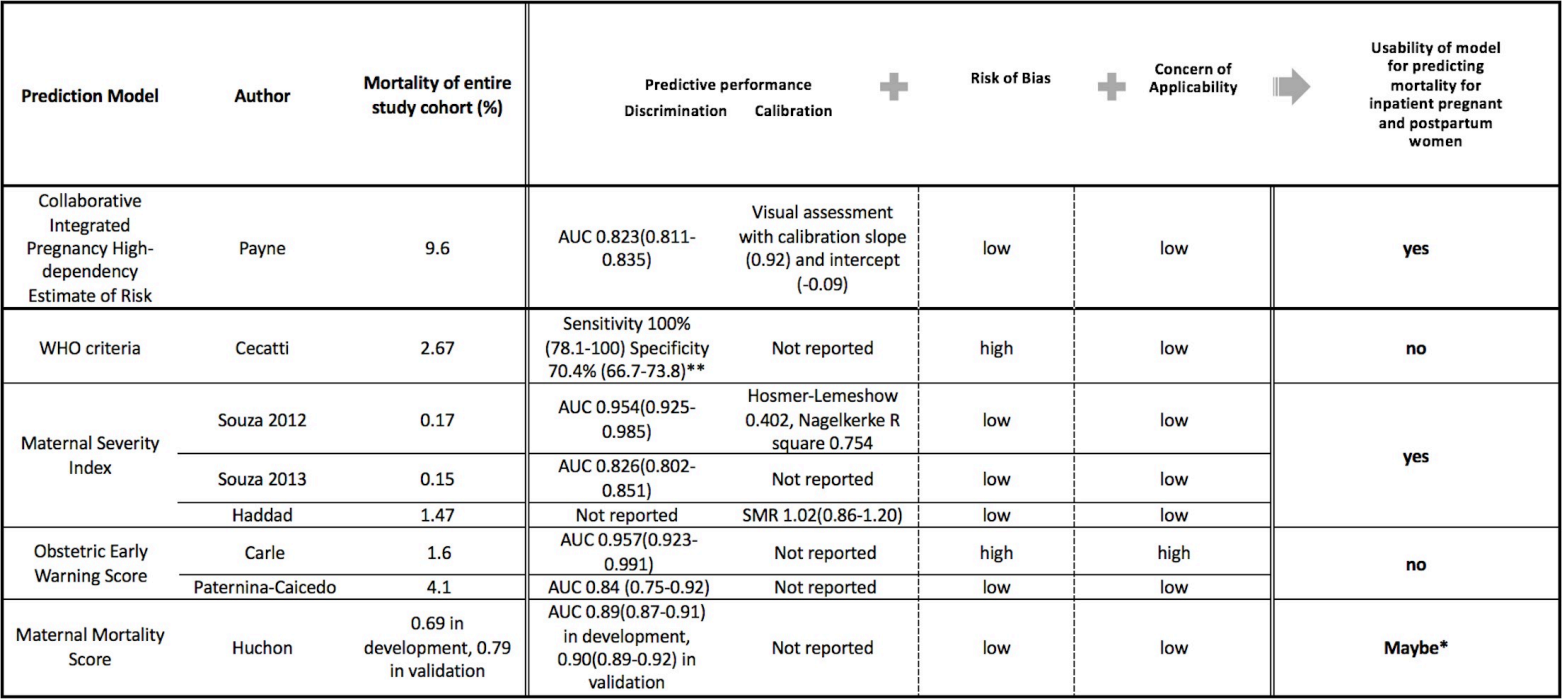
Characteristics of predictive models originally developed from obstetric populations. *PROBAST does not currently provide a “Maybe” option; however, we have added this term for perceived intermediate/potentially usable models for pregnant and post-partum populations. **for at least one of the WHO criteria.

**Fig 4:**
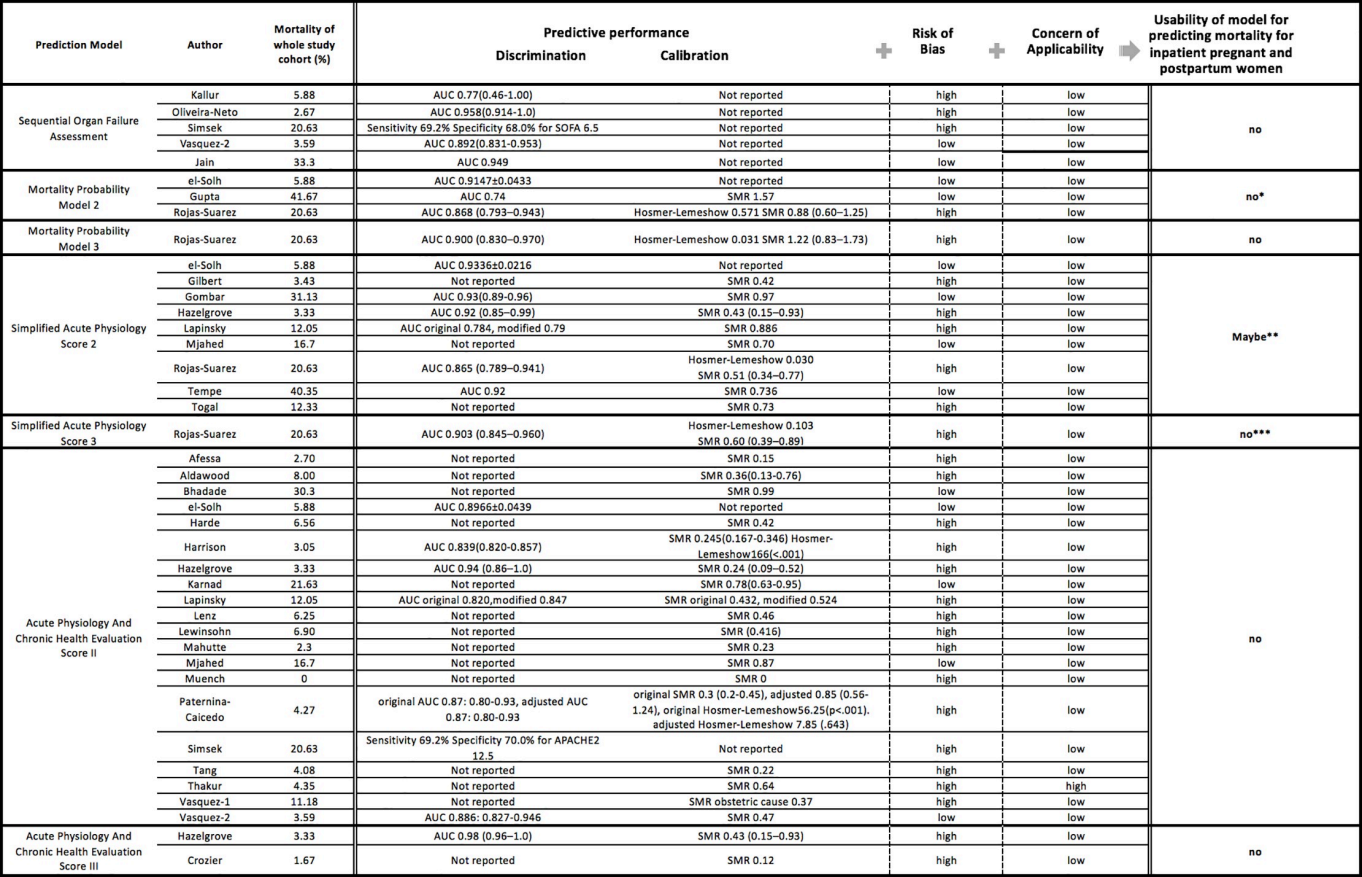
Characteristics of predictive models originally developed from non-obstetric population. * Results were inconsistent across the full number of studies examining this model. ** Sensitivity analysis showed good discrimination and calibration, although results were inconsistent across the full number of studies examining this model. ***Due to concern of a high risk of bias due to a low SMR.

Our initial search of MEDLINE, EMBASE and Scopus retrieved 9,710 citations, 8,935 of which remained after removal of duplications (Fig 2). Screening of titles and abstracts resulted in 74 relevant articles for which the manuscript full text was assessed for final eligibility. An additional 11 articles were identified through the reference lists and citation tracking of 316 relevant articles with use of Web of Science. Finally, 38 studies of 12 prediction models met inclusion criteria (Tables 1 and 2, Figs 3 and 4, and S1 Table) [4,17,36–45,28,46–55,29,56–63,30–35]. The TRIPOD checklist of all 38 eligible articles is available on request (S2 File).

**Table 1:** The number and setting of eligible studies in each prediction model, and original population and setting for development of each study.

	Original patient population	Original setting	External Validation in another Obstetric population (number of studies)
Acute Physiology And Chronic Health Evaluation score II & III*	Non-obstetric	ICU	21
Simplified Acute Physiology Score 2 & 3	Non-obstetric	ICU	9
Sepsis-related Organ Failure Assessment	Non-obstetric	ICU	5
Mortality Prediction Model 2 & 3	Non-obstetric	ICU	3
World Health Organization criteria	Obstetric	ICU	1
Obstetric Early Warning Score	Obstetric	ICU	1
Collaborative Integrated Pregnancy High-dependency Estimate of Risk	Obstetric	ICU	0
Maternal Severity Index	Obstetric	General ward	2
Maternal Mortality Score	Obstetric	General ward **	0

ICU = intensive care unit

* include updated tool from original model

** Hospitals in developing countries

**Table 2:** Summary of 38 eligible articles.

Prediction models	Country	Study design	Study period	Study population	Sample size	Outcomes
Collaborative Integrated Pregnancy High-dependency Estimate of Risk [Payne]	17 countries	Retrospective cohort*	2000–12	ICU	477	Antepartum and postpartum mortality
Combined WHO criteria: laboratory and management criteria [Cecatti]	Brazil	Retrospective cohort*	2002–7	All hospitalization	673	ICU mortality
Maternal Severity Index [Souza]*****	Brazil	Prospective cohort**	2009–10	All hospitalization	82,388	Hospital mortality
Maternal Severity Index [Souza]	29 countries	Cross-sectional*	2010–11	All hospitalization	314,623	Hospital mortality
Maternal Severity Index [Haddad]*****	Brazil	Cross-sectional*	2009–10	All hospitalization	9,555	Hospital mortality
Obstetric early warning score, Modified Early Obstetric Warning System, the confidential enquiries into maternal death Obstetric EWS, the royal college of physician's non-obstetric NEWS [Carle]****	United Kingdom	Retrospective cohort**	1995–2008	ICU	4,440	ICU mortality
Obstetric early warning score [Paternina-Caicedo]	Colombia	Retrospective cohort*	2006–11	ICU	702	Antepartum and postpartum mortality
Maternal mortality Score [Huchon]	Senegal and Mali	Prospective cohort**	2007–8	All hospitalization	43,624 for development, 46,328 for validation	Hospital mortality
SOFA [Kallur]	India	Retrospective cohort*	2011–12	ICU	69	Mortality ***
SOFA [Oliveira-Neto]	Brazil	Retrospective cohort*	2002–7	ICU	673	ICU mortality
SOFA [Jain]	India	Prospective cohort*	2010–11	ICU	90	ICU mortality
APACHE II, SOFA [Simsek]	Turkey	Retrospective cohort*	1999–2009	ICU	63	ICU mortality
APACHEII, SOFA [Vasquez]	Argentina	Prospective cohort*	2012	ICU	362	Hospital mortality
APACHE II, SAPS2, MPM2 [el-Solh]	United States	Retrospective cohort*	1989–95	ICU	93	Antepartum and postpartum mortality
MPM2 [Gupta]	India	Retrospective cohort*	2009–10	ICU	24	ICU mortality
SAPS2, 3 MPM2, 3 [Rojas-Suarez]	Colombia	Retrospective cohort*	2006–11	ICU	726	Mortality ***
APACHE II, SAPS2, APACHE III [Hazelgrove]	England	Retrospective cohort*	1994–6	ICU	210	Mortality ***
SAPS2 [Gombar]	India	Retrospective cohort*	2007–12	ICU	151	Mortality ***
APACHE II, SAPS2 [Lapinsky]	6 countries	Retrospective cohort*	1994–8	ICU	332	Hospital mortality
APACHE II SAPS2 [Mjahed]	Morocco	Retrospective cohort*	1995–2002	ICU	364	Hospital mortality
SAPS2 [Gilbert]	United States	Cohort, unknown pro/retro*	1991–1998	ICU	233	Hospital mortality
SAPS2 [Tempe]	India	Retrospective cohort*	2002–04	ICU	57	Hospital mortality
SAPS2 [Togal]	Turkey	Retrospective cohort*	2006–09	ICU	73	Mortality ***
APACHE II [Afessa]	United States	Retrospective cohort*	1991–1998	ICU	74	Hospital mortality
APACHE II [Aldawood]	Saudi Arabia	Retrospective cohort*	1999–2009	ICU	75	ICU mortality
APACHE II [Bhadade]	India	Prospective cohort*	2009–10	ICU	122	ICU mortality
APACHE II [Harde]	India	Prospective cohort*	2011–12	ICU	61	Mortality ***
APACHE II [Harrison]****	United Kingdom	Retrospective cohort*	1995–2003	ICU	1,902	Hospital mortality
APACHE II [Karnad]	India	Retrospective cohort*	1997–2001	ICU	453	Mortality ***
APACHE II [Lenz]	Austria	Retrospective cohort*	March 1996- Oct 2001, Nov 2004-Jun 2005	ICU	80	Hospital mortality
APACHE II [Lewinsohn]	Israel	Retrospective cohort*	non specific 8 years	ICU	58	Hospital mortality
APACHE II [Mahutte]	Canada	Retrospective cohort*	1992–97	ICU	131	ICU mortality
APACHE II [Muench]	United States	Prospective cohort*	Non specific 2 years	ICU	34	Mortality ***
APACHE II [Tang]	China	Retrospective cohort*	1998–1995	ICU	49	ICU mortality
APACHE II [Thakur]	United States	Retrospective cohort*	2006–12	ICU	69	ICU mortality
APACHE II, updated APACHE II [Paternina-Caicedo]	Colombia	Retrospective cohort*	2006–11	ICU	654	ICU mortality
APACHE II [Vasquez]	Argentina	Retrospective cohort*	1998–2005	ICU	161	ICU mortality
APACHE III [Crozier]	Australia	Retrospective cohort*	2006–8	ICU	60	Hospital mortality

Abbreviations: WHO: World Health Organization, SOFA: Sequential Organ Failure Assessment, APACHE: Acute Physiology And Chronic Health Evaluation, SAPS: Simplified Acute Physiology Score, MPM: Mortality Probability Model

* validation study

** development and validation study

*** without specific time period specified

**** Same cohort from ICNARC (Intensive Care National Audit and Research Center in United Kingdom)

*****Same cohort from The Brazilian Network for Surveil- lance of Severe Maternal Morbidity

Of these 38, 4 studies both developed *and* validated their model (Table 1) [30,37,54,63], and the remainder (n = 34) were primarily validation studies. Most studies (n = 36) employed a cohort design (prospective in 7, retrospective in 28 and unknown in 1) and 2 used a cross-sectional design (Table 2). Nine studies investigated more than one prediction model in the study. Samples size in included studies ranged from 24 to more than 80,000 subjects. The most commonly reported primary outcome was hospital mortality (n = 14), followed by ICU mortality (n = 13), although eight studies did not specify timing or place of death. Mortality varied across the studies, with an average rate 10.4%, ranging from 0 to 41.7% (Table 2, Fig 3 and Fig 4). Most studies (n = 16) were from a single developed country, 4 were multi-country studies, and 18 were from developing countries.

No uniform measure was reported to quantify the predictive performance (ability to predict outcomes of interest) of eligible models (Fig 3 and Fig 4). Among 20 studies reporting AUC, values ranged from 0.77 to 0.98; 18 studies reported AUCs higher than 0.8, indicating good discriminative performance. SMR was the most commonly reported form of calibration (n = 26), ranging from 0 to 1.57. Only 4 studies reported the Hosmer-Lemeshow goodness-of-fit test [50,53,54,61]. Four studies investigated classification measures (e.g. sensitivity and specificity) for a particular cut point of each model with or without reporting discrimination and calibration [4,31,38,43] (Fig 3 and Fig 4).

The methodological quality for each study as determined by PROBAST (Fig 3 and Fig 4, and S3 File) is summarized by a measure of model *Usability* [25] [Fig 1].

### Predictive models originally developed from obstetric populations

**Fig 5:**
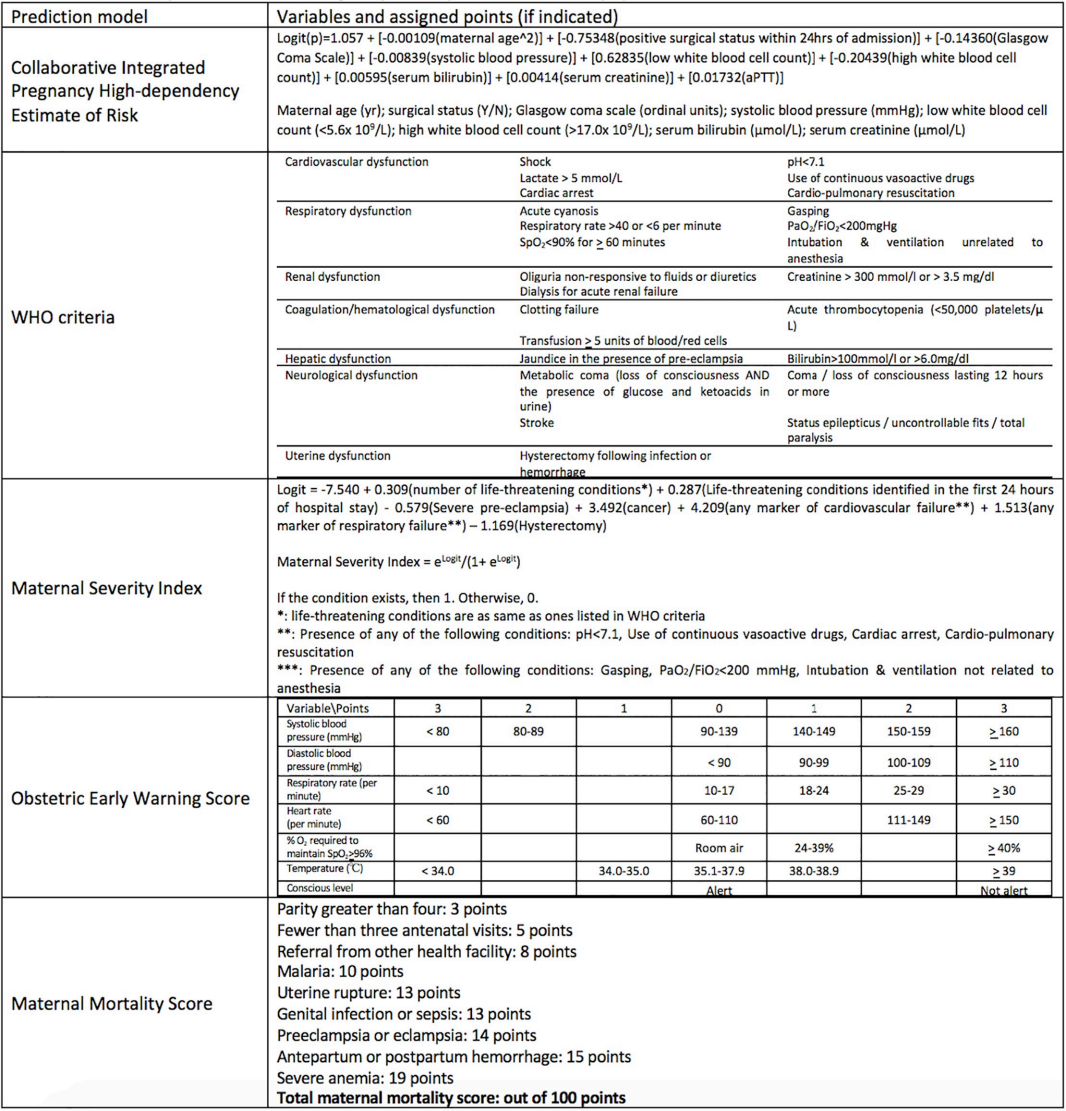
Box summary of variables of predictive models originally developed from obstetric populations.

Five models were developed and validated to identify obstetric patients at risk of death using a combination of comorbid health conditions, clinical characteristics, physiological and laboratory based data: the Collaborative Integrated Pregnancy High-dependency Estimate of Risk (CIPHER), the World Health Organization (WHO) Criteria, Maternal Severity Index, Obstetric Early Warning Score and the Maternal Mortality Score [30,31,37,54,63] (Table 2, Figs 3 and 5).

The Collaborative Integrated Pregnancy High-dependency Estimate of Risk was developed for predicting mortality and prolonged organ support of pregnant and postpartum women. The cohort included individuals in Intensive Care Units of 11 high-, middle- and low-income countries, with an overall mortality rate 9.6% [63]. The final model contains 10 predictors: maternal age, surgery in the preceding 24 hours, systolic blood pressure, Glasgow Coma Scale, serum bilirubin, activated partial thromboplastin time, serum creatinine, potassium, sodium and arterial blood gas pH. Discrimination (AUC: 0.823, 95% CIs 0.811–0.835) and calibration (Graphic plot [intercept—0.09, slope 0.92]) were internally validated with a bootstrapped sample—in a graphic plot, perfect calibration shows a slope of 1 and intercept of 0. There was no external validation.

The WHO introduced criteria consisting of patient characteristics, vital signs, and physiological and laboratory data to predict maternal severe morbidity. Cecatti et al assessed laboratory- and management-based markers of severity of illness (for example, use of vasopressors, dialysis, ventilation, transfusion, need for hysterectomy, receipt of cardiopulmonary resuscitation) on the risk of maternal death [31]. Using a combined count of all events, sensitivity and specificity (with at least one criteria of severe morbidity) for predicting death was assessed. Mortality rate of the cohort was 2.67%. Subsequently, Souza et al. validated the WHO criteria’s predictive ability using a different dataset in a distinct middle-income country [54]. The Maternal Severity Index was developed on the basis of this validation, incorporating the WHO criteria in addition to other markers of severe illness, for those who were in general wards and also Intensive Care Units [54]. The Maternal Severity Index was subsequently externally validated using multi-national cohorts, demonstrating a good AUC (0.82, 95% CIs 0.78–0.86) and SMR (1.02) [29,49]. In total, 3 studies investigated the Maternal Severity Index, with mortality ranging from 0.15 to 1.47%.

The Obstetric Early Warning Score incorporates vital signs, level of consciousness and oxygen requirements. It was developed and internally validated in the United Kingdom with mortality 1.6% and an AUC of 0.957 (95% CIs 0.923–0.991) for predicting death [30]. The study was considered to be at high risk of bias due to a number of excluded participants from the final model. There was high concern of applicability to the obstetric patient population in general wards because the model was developed on the basis of ICU patients and mortality in ICU rather than hospital patients and hospital mortality. Recently, an external validation was performed, demonstrating good predictive ability (AUC: 0.84, 95% CIs 0.75–0.92) for mortality among obstetrics patients, with a mortality rate 4.1% [61]. However, calibration has not yet been evaluated.

The Maternal Mortality Score uses 9 clinical and social conditions, was developed in low- and middle-income countries, and showed good discrimination ability in the development (AUC 0.89, 95% CIs 0.87–0.91, mortality rate: 0.69%) and validation (AUC 0.90, 95% CIs 0.89–0.92, mortality rate: 0.79%) cohorts [37]. The Maternal Mortality Score has only been validated by one study, and calibration has not yet been evaluated.

Of all models developed and validated to identify obstetric patients at risk of death using a combination of comorbid health conditions, clinical characteristics, physiological and laboratory based data, the Collaborative Integrated Pregnancy High-dependency Estimate of Risk and the Maternal Severity Index were developed/validated from studies with a low risk of bias and low concern of applicability of the model to obstetric populations for predicting maternal death, leading to a designation of high “usability” [29,37,54,63] (Fig 3).

### Models originally developed primarily from non-obstetric patient populations

**Fig 6:**
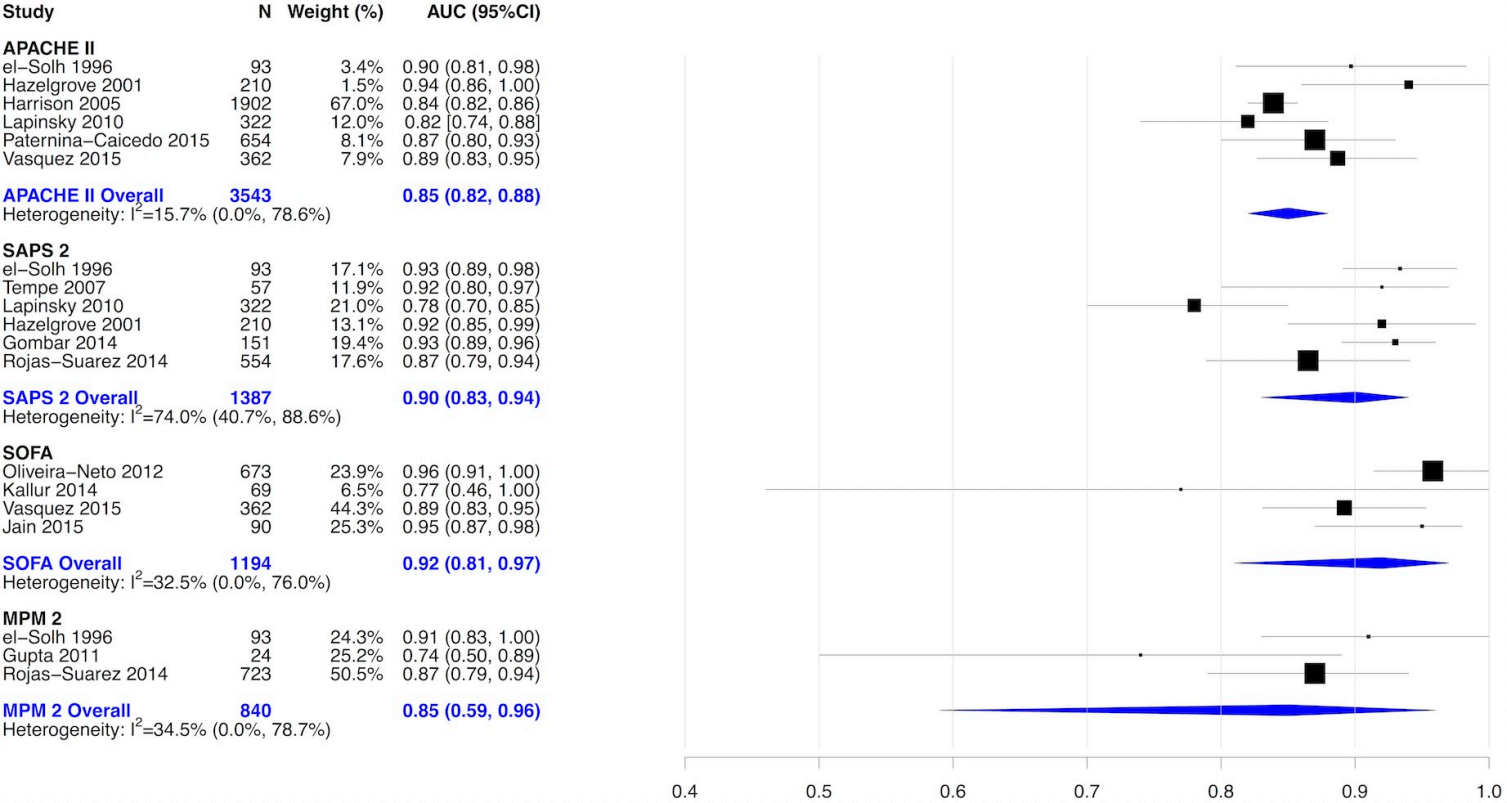
Pooled AUC of APACHE II, SAPS, SOFA and MPM2.

Thirty studies explored 7 predictive models developed primarily from non-obstetric patient populations—the Simplified Acute Physiology Score 2 and 3, Acute Physiology and Chronic Health Evaluation Score 2 and 3, Sequential Organ Failure Assessment, and the Mortality Probability Model, versions 2 and 3) [4,17,40–49,28,50–53,55–57,59,60,62,32–36,38,39] (Fig 4)—that were initially developed from non-obstetric critically ill patient populations and incorporated *patient characteristics*, *comorbidities*, *physiological and laboratory-based data*. The AUC across these 7 models was near 0.80, demonstrating good discriminative ability. Most studies (n = 25) reported the standardized mortality ratio for calibration, which varied from 0 to 1.57, indicating that some models under-estimate and others over-estimate true mortality (Fig 4). Six studies of the Acute Physiology and Chronic Health Evaluation II [4,17,32,36,50,53], 6 studies of the Simplified Acute Physiology Score 2 [17,32,33,36,42,45], 4 studies of the Sequential Organ Failure Assessment [4,38,41,60] and 3 studies of the Mortality Probability Model [32,34,42] were pooled in separate meta-analyses [Fig 6]. SOFA had the highest discrimination followed by SAPS2 and APACHE II and MPM2. None of models originally developed primarily from non-obstetric patient populations had a designation of high “usability”.

### Sensitivity analyses

A preplanned sensitivity analysis was performed, among 9 studies with low risk of bias and low concern of applicability [4,32–34,39,45,57,58,60]. Five studies evaluated APACHE II [4,32,39,57,58], 4 studies evaluated SAPS 2 [32,33,45,57], 2 studies evaluated SOFA [4,60] and 2 studies evaluated MPM 2 [32,34]. APACHE II showed good discrimination, but over-estimated death. The MPM 2 showed good discrimination, but underestimated death. SOFA showed good discrimination, but calibration was not investigated. AUCs of SAPS 2 were pooled in this sensitivity analysis. The SAPS 2 summary AUC was 0.84 (95% CIs [0.92–0.94] and I^2^ = 0%) with good discrimination, and calibration [S1 Fig].

A *post-hoc* sensitivity analysis was conducted according to mortality rates. AUCs were not pooled in this analysis because of the limited number of studies for each model. In studies from countries with low mortality–less than 1%—the Maternal Severity Index is the only model that is externally validated. In studies from countries with moderate mortality rates–less than 10%—all predictive models originally developed from non-obstetric population, except SAPS3, were investigated. However, the CIPHER model is the only well validated model for both discrimination and calibration. Only predictive models developed from non-obstetric population were investigated in studies with high and very high mortality. Predictive performance (i.e. discrimination and calibration) of models in studies with high and very high mortality were inconsistent across studies.

### Subgroup analyses

Two *post-hoc* subgroup analyses were carried out. In predictive models developed from obstetric population, the CIPHER model and the Maternal Severity Index were investigated in both high- and low-income countries. Studies for predictive models developed from non-obstetric populations were well balanced between high- and low-income countries, and predictive performance seemed similar. The Maternal Severity Index was the only model that was investigated in general obstetric ward patients; therefore, it was difficult to estimate how other settings might affect predictive performance of the models.

## Discussion

### Main findings

This systematic review identified 38 studies that developed and/or validated 12 models for predicting mortality among hospitalized pregnant and postpartum women. The Collaborative Integrated Pregnancy High-dependency Estimate of Risk (CIPHER) for hospitalized critically ill obstetric populations, and the Maternal Severity Index for hospitalized general obstetric populations have good discrimination, calibration, were developed from studies with a low risk of bias and internally and/or externally validated for critically ill pregnant and postpartum women. Prediction models developed from non-obstetric patients and from general ICU patient populations have very good discrimination but are at risk of over- or under-estimation of true mortality.

### Interpretations

Maternal death is rare event. Hence, predictive models for maternal death seem to show high discriminative performance. In this context, calibration is important in assessing overall predictive performance. However, most of eligible studies in this review reported SMR, which is known to be a relatively crude measure of calibration [18] and ideally should be considered across the full range of outcome rates [64]. The Hosmer-Lemeshow goodness-of-fit test is very sensitive to sample size and also therefore an imperfect measure of calibration [65]. Therefore, calibration in models reporting SMR and Hosmer-Lemeshow goodness-of-fit test need to be interpreted in the context of the setting of the study, in addition to the setting of its application.

The wide range of mortality of the eligible 38 studies helps to explain high heterogeneity we found for various prediction models, across the studies. Also, the studies were assessed in different clinical settings and among patients of varying initial severity of illness, making comparability of predictive performance challenging. For example, a predictive model developed in a country where mortality is high might falsely under- or over-estimate mortality in a country where mortality is much lower, or vice versa. On the other hand, the average mortality among studies of predictive models originally developed from obstetric populations was higher than one from non-obstetric populations. This might explain why the calibration among studies of predictive models from non-obstetric populations appears to be worse than one from obstetric populations.

### Implications

In observational studies, a risk adjustment tool is essential to help take into account characteristics (e.g. the severity of a patient’s illness) that may influence or confound an attempt to estimate the magnitude and significance of new factor of interest on a *cohort of patients’* clinical outcome such as death. Both CIPHER and the Maternal Severity Index models might also be used to help risk adjust mortality differences between health facilities as part of quality assurance and improvement initiatives [29,54,63].

However, none of the indices studied have sufficient predictive ability to be used in determining the outcome of an *individual* obstetric patient. A risk prediction model revealing a 90% risk of death in a selected population of 100 pregnant or post-partum women cannot differentiate which *individual* 10 women will survive and which will die. That is, there is a risk of underestimating risk in low-risk patients, and of overestimating risk in high-risk patients. Therefore, estimates based on risk prediction models should not directly affect the decisions for withdrawing or withholding management of individual seriously ill pregnant or postpartum women. However, despite uncertainty of predictive performance in assessing individual risk, prediction models may help to identify pregnant and peri-partum women at high risk of critical illness or death and stimulate increased monitoring or preventive measures.

Risk prediction models developed from non-obstetric patient populations should generally not be applied to obstetric patient populations, *if a better alternative exists*. The CIPHER model for hospitalized critically ill obstetric populations, and the Maternal Severity Index for hospitalized general obstetric populations are suggested for use, when sufficient data exists. In low/middle income countries, because of the large number of variables required for the models, feasibility is a concern, and therefore, the Maternal Mortality Score may be more appropriate. For future research, investigation of few different prediction models within the same population is the ideal study design, to determine the best model for predicting maternal mortality.

### Strengths and limitations

This systematic review has a number of strengths. This is the first systematic review of risk prediction models for maternal mortality in obstetric populations. We followed the most recent guideline of systematic reviews for risk prediction models [18] and the PRISMA-P 2015 statement (S4 File) [19]. We employed a formal and broad search strategy, without language restriction and differentiated between risk prediction models for obstetric and non-obstetric populations. We used a robust tool to identify studies at risk of bias using the Prediction model study Risk Of Bias Assessment Tool [25], which allowed us to conduct sensitivity analyses on the most applicable studies for obstetric populations. Next, we have developed our systematic review according to the ROBIS guidelines for detecting bias in systematic reviews (S5 File) [66]. Lastly, our findings are applicable to pregnant and postpartum women who are admitted to either ICUs or acute care wards of general hospitals.

This systematic review also has certain limitations. First, individual patient data from each prediction model evaluation was not available, which precludes an opportunity re-calculate model performance characteristics, and precludes an opportunity to meta-analyze some metrics we evaluated. Second, the data sources used in the various evaluations were derived from diverse clinical settings across and within countries where patient characteristics and clinical practice vary. Yet, this clinical heterogeneity of included studies allows us to make inferences across diverse settings, for patients in an ICU or a hospital. Third, the eligible studies reported mortality at different measurement points (e.g. ICU mortality, hospital mortality), which was challenging to meta-analyze. Fourth, some risk prediction models we identified in the current study could perform differently in certain common diseases of maternal death (e.g. infection) [67], although our findings are likely applicable to most general obstetric populations. Fifth, physiology-based predictions models developed from non-obstetric populations may be challenging to apply to pregnant patients because of changes in physiology (heart rate, blood pressure, respiratory rate for example) that occur as a usual course of pregnancy. Importantly, there were a limited number of studies validating prediction models developed from obstetric population.

## Conclusion

Mortality risk prediction models developed from obstetric patient populations, such as the Collaborative Integrated Pregnancy High-dependency Estimate of Risk (CIPHER) model and the Maternal Severity Index, have good discrimination and calibration, developed/validated from studies with a low risk of bias, and should be encouraged for use in prospectively designed studies, trials and quality improvement research among critically ill pregnant and postpartum women. While prediction models previously developed from general and non-obstetric patient populations such as the APACHE, MPM, SAPS, and SOFA scores are at some risk of over- or under-estimating mortality, they generally have good discrimination and may reasonably be used when pregnancy-specific models are unavailable.

## Supporting information

S1 Table Explanation for excluded studies while full text assessment. (PDF)

S1 Fig Sensitivity analysis, pooled AUC of SAPS 2. (TIFF)

S1 File Search strategy for MEDLINE, EMBASE, PubMed and web of science. (DOCX)

S2 File Agreed TRIPOD checklist for all eligible 38 studies. (XLSX)

S3 File Agreed risk of bias assessment for all eligible 38 studies (PROBAST). (XLSX)

S4 File PRISMA checklist. (DOC)

S5 File ROBIS checklist. (PDF)

## Abbreviations

AUC: area under the receiver operating characteristic curve
SAPS: simplified acute physiology score
APACHE: acute physiology and chronic health evaluation score
MPM: the mortality prediction model
SOFA: sequential organ failure assessment
ICU: intensive care unit
PRISMA: the Preferred Reporting Items for Systematic Reviews and Meta-Analyses protocols
CI: confidence interval
SE: standard error
SMR: Standardized Mortality Ratio
TRIPOD: Transparent Reporting of a multivariable prediction model for Individual Prognosis Or Diagnosis
PROBAST: Prediction model study Risk Of Bias Assessment Tool
WHO: World Health Organization
