# Supplementary material for: Risk prediction models for maternal mortality: A systematic review and meta-analysis
Source: PLoS One. 2018 Dec 4;13(12):e0208563. doi: 10.1371/journal.pone.0208563 (PMC6279047; doi:10.1371/journal.pone.0208563)
Supplement: S1 Table — (PDF) [file pone.0208563.s001.pdf]

Author	Title	Journal	Year	Reason for exclusion
Adeniran, A. S., Bolaji, B. O., Fawole, A. A. and Oyedepo, O. O.	Predictors of maternal mortality among critically ill obstetric patients	Malawi Medical Journal	2015	No Performance of Models Reported
Ahmed,N. K. A.;Makki,M.;Mahmoud,S.;Amu,O.	Maternal near-miss in a UK district hospital: 5 year review of critical care admissions	BJOG: An International Journal of Obstetrics and Gynaecology	2013	Conference abstract
Ashraf,N.;Mishra,S. K.;Kundra,P.;Veena,P.;Soundaraghavan,S.;Habeebullah,S.	Obstetric patients requiring intensive care: A one year retrospective study in a tertiary care institute in India	Anesthesiology Research and Practice	2014	No Performance of Models Reported
Bateman,B. T.;Mhyre,J. M.;Hernandez-Diaz,S.;Huybrechts,K. F.;Fischer,M. A.;Creanga,A. A.;Callaghan,W. M.;Gagne,J. J.	Development of a comorbidity index for use in obstetric patients	Obstetrics and gynecology	2013	No models investigated or comorbidity indexes
Bhadade,R.;De'Souza,R.;More,A.;Harde,M.	Maternal outcomes in critically ill obstetrics patients: A unique challenge	Indian Journal of Critical Care Medicine	2012	No Performance of Models Reported
Bhagwanjee,S.;Paruk,F.;Moodley,J.;Muckart,D. J.	Intensive care unit morbidity and mortality from eclampsia: an evaluation of the Acute Physiology and Chronic Health Evaluation II score and the Glasgow Coma Scale score	Critical care medicine	2000	Specific disease
Bouvier-Colle,M.;Salanave,B.;Ancel,P. Y.;Varnoux,N.;Fernandez,H.;Papiernik,E.;Breart,G.;Benhamou,D.;Boutroy,P.;Caillier,I.;Dumoulin,M.;Fournet,P.;Elhassani,M.;Puech,F.;Poutot,C.	Obstetric patients treated in intensive care units and maternal mortality. Regional Teams for the Survey	European Journal of Obstetrics, Gynecology, & Reproductive Biology	1996	No Performance of Models Reported
Crozier,T. M.;Wallace,E. M.	Obstetric admissions to an integrated general intensive care unit in a quaternary maternity facility	Australian & New Zealand Journal of Obstetrics & Gynaecology	2011	No Performance of Models Reported
Czechowski,M.;Duda,I.;Gronska,E.	Critical care management and outcomes of obstetrics patients in intensive care unit	Intensive care medicine	2011	Conference abstract
Das Neves,A.;Vasquez,D. N.;Intile,D.;Cicora,F.;Saenz,M. G.;Loudet,C.;Canales,H.;Casanova,M.;Aphalo,V.;Balasini,C.;Scappellato,J. L.;Reina,R.;Savero,J.;Desmery,P.;Estenssoro,E.	Outcome and level of intervention of critically ill obstetric patients from the public health sector vs. the private health sector: Prospective cohort	American Journal of Respiratory and Critical Care Medicine	2011	Conference abstract
Demirkiran,O.;Dikmen,Y.;Utku,T.;Urkmez,S.	Critically ill obstetric patients in the intensive care unit	International Journal of Obstetric Anesthesia	2003	No Performance of Models Reported
Dennis,A.T.; Chambers,E.	Maternal morbidity: An analysis of high dependency unit care in pregnant or recently pregnant women	International Journal of Obstetric Anesthesia	2016	Conference abstract
El Ayadi, A. M.; Nathan,H.L.; Seed,P.T.; Butrick,E.A.; Hezelgrave,N.L.; Shennan,A.H.; Miller,S.	Vital Sign Prediction of Adverse Maternal Outcomes in Women with Hypovolemic Shock: The Role of Shock Index	PLOS ONE	2016	Specific disease
El-Ayadi, A., Nathan, H. L., Seed, P. T., et al.	Vital sign prediction of adverse maternal outcomes in women with hypovolemic shock: The role of shock index	PLOS ONE	2015	Specific disease
Eppes, C. S., Schupp, J. and Dildy, G.	Shock index: A potential criterion for a maternal early warning system	American Journal of Obstetrics and Gynecology	2016	Conference abstract
Friedman, A. M.	Maternal Early Warning Systems	Obstet Gynecol Clin N Am	2015	Review
Geller,S. E.;Rosenberg,D.;Cox,S. M.;Brown,M. L.;Simonson,L.;Driscoll,C. A.;Kilpatrick,S. J.	The continuum of maternal morbidity and mortality: Factors associated with severity	American Journal of Obstetrics and Gynecology	2004	Models not for predicting mortality
Geller,S. E.;Rosenberg,D.;Cox,S.;Brown,M.;Simonson,L.;Kilpatrick,S.	A scoring system identified near-miss maternal morbidity during pregnancy	Journal of clinical epidemiology	2004	Models not for predicting mortality
Gopal,G.;Gatongi,D. K.;Kamat,A.;Nicoll,A.	Maternal intensive care unit (ICU) admissions in obstetrics: Indications and outcome	Journal of Obstetrics and Gynaecology	2010	Conference abstract

Guleria,K.;Jain,S.;Ahuja,S.;Suneja,A.;Vaid,N. B.	Sequential organ failure assessment score for evaluating outcome in obstetric cases admitted to intensive care unit	BJOG: An International Journal of Obstetrics and Gynaecology	2014	Conference abstract
Hedriana,H.L.; Wiesner,S.; Downs,B.G.; Pelletreau,B.; Shields,L.E.	Baseline assessment of a hospital-specific early warning trigger system for reducing maternal morbidity	International Journal of Gynecology and Obstetrics	2016	Models not for predicting mortality
Heinonen,S.;Tyrvainen,E.;Saarikoski,S.;Ruokonen,E.	Need for maternal critical care in obstetrics: A population-based analysis	International Journal of Obstetric Anesthesia	2002	No Performance of Models Reported
Kainiemi,E.K.; Lavonen,L.	Need for intensive care for obstetric patients in Turku university hospital and in central Finland central hospital 2009-2013	Acta Anaesthesiologica Scandinavica	2015	Conference abstract
Kamal,E. M.;Behery,M. M. E.;Sayed,G. A. E.;Abdulatif,H. K.	RIFLE classification and mortality in obstetric patients admitted to the intensive care unit with acute kidney injury: A 3-year prospective study	Reproductive Sciences	2014	Specific disease
Kelebek Girgin,N.;Iscimen,R.;Otlar,B.;Kahveci,S. F.;Ozcan,B.	An analysis of obstetric patients treated in the intensive care unit. Turkish] Yogun bakim unitesinde tedaviedilen obstetrik olgularin analizi	Anestezi Dergisi	2006	Conference abstract
Khan,M. S.;Sultana,T.	Is delayed ICU referral associated with adverse maternal outcome? A study at tertiary level hospital in India	International Journal of Gynecology and Obstetrics	2012	Conference abstract
Lam,G.K.	Obesity and the Critical Care Pregnant Patient	Obstetrics and gynecology clinics of North America	2016	Review
Lapinsky,S. E.;Kruczynski,K.;Seaward,G. R.;Farine,D.;Grossman,R. F.	Critical care management of the obstetric patient	Canadian Journal of Anaesthesia	1997	No Performance of Models Reported
Lelong,E.;Pourrat,O.;Pinsard,M.;Goudet,V.;Badin,J.;Mimoz,O.;Pierre,F.	Admission of women to an intensive care unit during pregnancy or the postpartum period: circumstances and prognosis. A retrospective series of 96 cases]	Revue de Medecine Interne	2013	No Performance of Models Reported
Lenz-Gebhart,A.;Hamat,I.;Rabl,M.;Pateisky,N.;Lehner,R.	Morbidity and mortality of obstetric patients admitted to the ICU of a tertiary hospital	Archives of Gynecology and Obstetrics	2010	Conference abstract
Lima,H.M.; Carvalho,F.H.; Feitosa,F.E.; Nunes,G.C.	Factors associated with maternal mortality among patients meeting criteria of severe maternal morbidity and near miss	Int J Gynaecol Obstet	2017	No models investigated or comorbidity indexes
Lin,Y.;Zhu,X.;Liu,F.;Zhao,Y. Y.;Du,J.;Yao,G. Q.;Li,W. X.;Jia,X. J.	Analysis of risk factors of prolonged intensive care unit stay of critically ill obstetric patients: a 5-year retrospective review in 3 hospitals in Beijing]	Zhongguo Wei Zhong Bing Ji Jiu Yi Xue/Chinese Critical Care Medicine/Zhongguo Weizhongbing Jijiuyixue	2011	No Performance of Models Reported
Lomas,C.;Sacanell,J.;Roca,O.;Masclans,J. R.;Rello,J.	Obstetric patients requiring ICU admission. A fifteen-year review	Intensive care medicine	2010	Conference abstract
Lopes,A. P.;Reis,Z.;Rezende,C.;Brandao,A.;Januzzi,P.;Cabral,A. C. V.	Survival analysis in an obstetric intensive care unit, according diagnosis at admission	Journal of perinatal medicine	2011	Conference abstract
Main, E. K., Abreo, A., McNulty, J., et al.	Measuring severe maternal morbidity: validation of potential measures	American Journal of Obstetrics and Gynecology	2015	Models not for predicting mortality
Metcalfe A; Lix LM; Johnson JA; Currie G; Lyon AW; Bernier F; Tough SC.	Validation of an obstetric comorbidity index in an external population	BJOG: An International Journal of Obstetrics and Gynaecology	2015	No models investigated or comorbidity indexes
Michael Stephanou,A.	Validation of the modified early obstetric warning system (MEOWS)	Anaesthesia	2012	Models not for predicting mortality

Munnur,U.;Karnad,D. R.;Bandi,V. D. P.;Lapsia,V.;Suresh,M. S.;Ramshesh,P.;Gardner,M. A.;Longmire,S.;Guntupalli,K. K.	Critically ill obstetric patients in an American and an Indian public hospital: Comparison of case-mix, organ dysfunction, intensive care requirements, and outcomes	Intensive care medicine	2005	No Performance of Models Reported
Nair,M.; Kurinczuk,J.J.; Knight,M.	Establishing a National Maternal Morbidity Outcome Indicator in England: A Population-Based Study Using Routine Hospital Data	PLOS ONE	2016	Models not for predicting mortality
Ortega Carnicer,J.;Ambros Checa,A.;Fernandez-Medina Criado,V.;Alegria,Ruiz De;Larramendi,Ruiz De;Miguel,Diarte De;Torrubia Chalmeta,R.	Critical illness in pregnant patients. Spanish] Enfermedades criticas en pacientes obstetricas	Medicina Intensiva	1998	No Performance of Models Reported
Paxton,J. L.;Presneill,J.;Aitken,L.	Characteristics of obstetric patients referred to intensive care in an Australian tertiary hospital	Australian and New Zealand Journal of Obstetrics and Gynaecology	2014	No Performance of Models Reported
Perez Assef,A.;Acevedo Rodriguez,O.;Del Consuelo,Tamayo Gomez;Oviedo Rodriguez,R.	Characterization of obstetric patients with multiple organ failure in the intensive care unit of a Havana Teaching Hospital, 1998 to 2006	MEDICC Review	2010	No Performance of Models Reported
Stevens,T. A.;Carroll,M. A.;Promecene,P. A.;Seibel,M.;Monga,M.	Utility of Acute Physiology, Age, and Chronic Health Evaluation (APACHE III) score in maternal admissions to the intensive care unit	American Journal of Obstetrics and Gynecology	2006	No Performance of Models Reported
Toufiki,R.; Mouaffak,Y.; El Adib, A. R.; Amine,M.	Which index severity to use in obstetric medium? (Comparison of 4 scores)	Annals of Intensive Care.Conference: French Intensive Care Society, International Congress Reanimation	2016	Conference abstract
Vasquez,D. N.;Das Neves,A. V.;Aphalo,V. B.;Loudet,C. I.;Roberti,J.;Cicora,F.;Casanova,M.;Canales,H. S.;Intile,A. D.;Scapellato,J. L.;Desmery,P. M.;Estenssoro,E.	Health insurance status and outcomes of critically ill obstetric patients: A prospective cohort study in Argentina	Journal of critical care	2014	Models not for predicting mortality
Witteveen,T.; De Koning, I.; Bezstarosti,H.; Van,Den Akker; Van Roosmalen, J.; Bloemenkamp,K.W.	Validating the WHO Maternal Near Miss Tool in a high-income country	Acta Obstetricia et Gynecologica Scandinavica	2016	No Performance of Models Reported
You,W. B.;Chandrasekaran,S.;Sullivan,J.;Grobman,W.	Validation of a scoring system to identify women with near-miss maternal morbidity	American Journal of Perinatology	2013	Models not for predicting mortality
Zuckerwise,L.C.; Lipkind,H.S.	Maternal early warning systems-Towards reducing preventable maternal mortality and severe maternal morbidity through improved clinical surveillance and responsiveness	Seminars in Perinatology.	2017	Review
