# Supplementary figures and images for: Risk prediction models for maternal mortality: A systematic review and meta-analysis
Source: PLoS One. 2018 Dec 4;13(12):e0208563. doi: 10.1371/journal.pone.0208563 (PMC6279047; doi:10.1371/journal.pone.0208563)

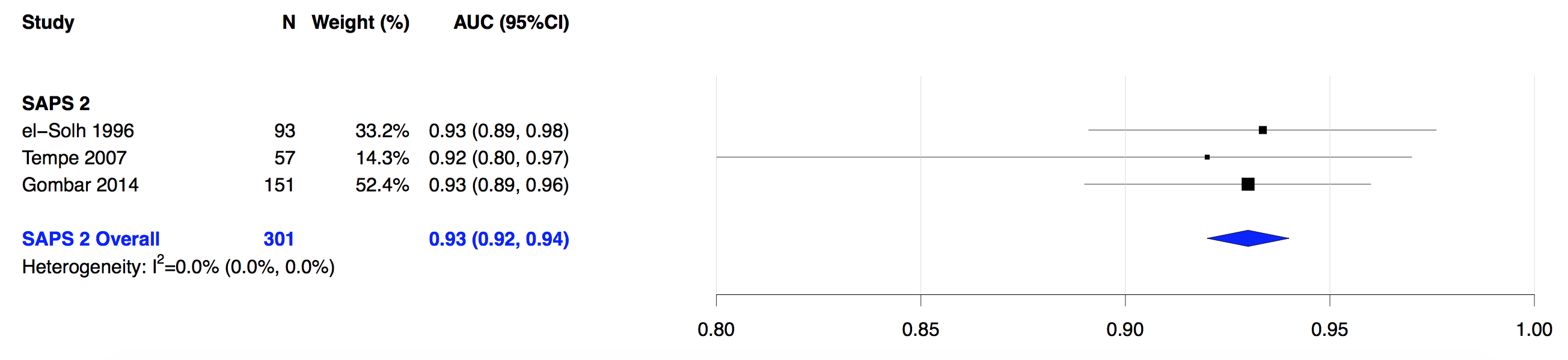

Supplement: S1 Fig — (TIFF) [file pone.0208563.s002.tiff]
