# Supplementary material for: Risk prediction models for maternal mortality: A systematic review and meta-analysis
Source: PLoS One. 2018 Dec 4;13(12):e0208563. doi: 10.1371/journal.pone.0208563 (PMC6279047; doi:10.1371/journal.pone.0208563)
Supplement: S1 File — (DOCX) [file pone.0208563.s003.docx]

S1 File. Search strategy for MEDLINE, EMBASE, PubMed and Web of Science

Maternal mortality index search strategies

Kazuyoshi Aoyama

Medline Search Strategy

Obstetrics/or Pregnant woman/or Postpartum period/or Perinatal care/ or exp Pregnancy/ or Pregnancy, high- risk/ or Exp Pregnancy complications/ or Delivery, Obstetric/ mo [mortality] or Labor, obstetric/ or Obstetric  labor complication/or Pregnan* or obstetric* or gestation*).tw. or (Postnat* or post-nat* or postpart* or post-part* or pre-nat* or prenat* or prepart* or pre-part*).tw. or Maternal care.tw. or Maternal health.tw.

AND

Maternal death/ or Maternal mortality/or Maternal adj5 death*.tw. or Maternal adj5 mortalit*.tw.

AND

Health status indicators/or apache/or organ dysfunction scores/ or patient acuity/ or “severity of illness index”/ or karnofsky performance statues/ or sickness impact profile/”outcome assessment (health care)” or Forecasting/ or Evaluation studies as topic/or program evaluation/ or “reproducibility of results/ or validation studies as topic/ or Exp Risk to include: risk/ or logistic models/ or risk assessment/ or risk factors/ or Calibration/ or decision support techniques/ or roc curve/ or SAPS.tw. or SOFA.tw. or MODS.tw. or MPM.tw. or Charlson.tw. or Elixhauser.tw. or APACHE.tw. or APACHE II.tw. or APACHE III.tw. or APACHE IV.tw. or (Acute physiology and chronic health evaluation).tw. or Mortality index.tw. or Mortality indices.tw. or receiver operating characteristic*.tw. or decision model*.tw. or prognostic model*.tw. or (predict* and risk*).tw. or predicting.tw. or (predictive adj1 (value of tests or model)).tw. or (prediction adj1 (model or tool or rule)).tw. or (risk adj1(assessment or score or engine or equation or algorithm or table or function)).tw. or (risk adj1 (calculator or calculation).tw. or (validation or discrimination or calibration).tw.

NOT exp animal/ not human

Embase Search Strategy

maternal mortality/ or maternal death/ or ((Maternal adj5 death*) or (Maternal adj5 mortalit*)).tw.

AND

(SAPS or SOFA or MODS or MPM or Charlson or Elixhauser or APACHE or APACHE II or APACHE III or APACHE IV or "Acute physiology and chronic health evaluation" or Mortality index or Mortality indices or receiver operating characteristic* or decision model* or prognostic model* or (predict* and risk*) or predicting or (predictive adj1 (value of tests or model)) or (prediction adj1 (model or tool or rule)) or (risk adj1 (assessment or score or engine or equation or algorithm or table or function)) or (risk adj1 (calculator or calculation)) or (validation or discrimination or calibration)).tw. or exp health status indicator/ or exp forecasting/ or program evaluation/ or evaluation study/ or risk/ or risk assessment/ or risk factor/ or statistical model/ or exp receiver operating characteristic/ or predictive value/ or calibration/

AND

obstetrics/ or pregnant woman/ or postnatal care/ or maternal care/ or perinatal care/ or exp pregnancy/  or high risk pregnancy/ or exp pregnancy complication/ or maternal care.ti,ab. or maternal health.ti,ab.  or  exp puerperium/ or exp postnatal care/ or perinatal care/ or exp delivery/  or exp labor/ or exp labor complication/ or (Pregnan* or obstetric* or gestation* or (Postnat* or post-nat* or postpart* or post-part* or pre-nat* or prenat* or prepart* or pre-part*)).tw. or (puerperium or labo?r or labo?rs or labo?ring).tw.

NOT exp animal/ not human/

PubMed Search Strategy

(obstetrics OR “pregnant woman” OR “postpartum period” OR “perinatal care” OR pregnancy OR “pregnancy complications” OR labor) and (“maternal death” OR “maternal mortality”) and (health status indicators OR forecasting OR evaluation studies OR program evaluation OR reproducibility of results OR validation studies as topic OR risk OR logistic models OR risk assessment OR risk factors OR calibration OR decision support techniques OR roc curve OR saps OR sofa OR mods OR MPM OR charlson OR elixhauser OR apache OR apache ii OR apache iii OR apache iv OR acute physiology and chronic health evaluation OR mortality index OR mortality indices OR receiver operating characteristic* OR decision model* OR prognostic model* OR (predict* and Risk*) OR predicting OR ((predictive OR prediction) adj1 (value of tests OR model OR tool OR rule)) OR (risk adj1 (assessment OR score OR engine OR equation OR algorithm OR table OR function OR calculator OR calculation))) and (publisher[sb] or inprocess[sb])

Web of Science Search Strategy

TS=(health status indicators OR forecasting OR "evaluation studies" OR "program evaluation" OR "reproducibility of results" OR "validation studies as topic" OR "logistic models" OR calibration OR "decision support techniques" OR "roc curve" OR saps OR sofa OR mods OR MPM OR charlson OR elixhauser OR apache OR "apache ii" OR "apache iii" OR "apache iv" OR "acute physiology" and "chronic health evaluation" OR "mortality index" OR "mortality indices" OR "receiver operating characteristic*" OR decision model* OR prognostic model* OR (predict* and Risk*) OR predicting OR ((predictive OR prediction) adj1 (value of tests OR model OR tool OR rule)) OR (risk adj1 (assessment OR score OR engine OR equation OR algorithm OR table OR function OR calculator OR calculation)))

AND

TS=(obstetrics OR pregnant OR postpartum OR perinatal OR pregnancy OR labor)

AND

TS=((maternal death) or (maternal mortality) or (maternal w/5 death*) or (maternal w/5 mortalit*))
